# Enhanced national surveillance of severe acute respiratory infections (SARI) within COVID-19 surveillance, Slovenia, weeks 13 to 37 2021

**DOI:** 10.2807/1560-7917.ES.2021.26.42.2100937

**Published:** 2021-10-21

**Authors:** Irena Klavs, Mojca Serdt, Veronika Učakar, Marta Grgič-Vitek, Mario Fafangel, Maja Mrzel, Lina Berlot, Uroš Glavan, Marjana Vrh, Tanja Kustec, Miha Simoniti, Božena Kotnik Kevorkijan, Tatjana Lejko Zupanc, the EPISARI Network, Members of the EPISARI Network, Members of the EPISARI Network, Irena Klavs, Mojca Serdt, Lina Berlot, Uroš Glavan, Tanja Kustec, Veronika Učakar, Marta Grgič-Vitek, Mario Fafangel, Maja Mrzel, Marjana Vrh, Tina Kaparić Kersnik, Marinka Krumpestar, Mojca Savnik Iskra, Miha Simoniti, Matej Breznar, Andrej Bartolić, Debora Kocijančič, Vesna Kovačič, Petra Falabella, Tanja Ribič Vidovič, Natalija Galinec, Jani Dernič, Teja Perenič Mamilovič, Matej Dolenc, Ana Ščavničar, Emil Pal, Jerneja Farkaš, Nina Žižek, Tatjana Remec, Primož Brkić, Eva Miler Mojškerc, Mihaela Slemnik, Anja Potočnik, Nina Kokalj, Rok Šater, Katja Kalan Uštar, Aleš Rozman, Lidija Studen, Jana Čelhar, Viktor Švigelj, Andreja Pečnik, Jasna Dobelšek Furst, Katja Jarm, Sonja Tomšič, Dejan Bregar, Anže Mihelič, Nataša Vuga, Metka Velušček, Tanja Cebin Skale, Andreja Uršič, Kristina Kržišnik, Mateja Matvoz Kos, Metka Vidovič, Valentina Winkler Skaza, Renata Nagode, Barbara Bitežnik, Tatjana Lejko Zupanc, Nina Žakelj, Katarina Lakner, Božena Kotnik Kevorkijan, Andraž Jug, Jožica Peterka Novak, Urška Zupanc

**Affiliations:** 1 Communicable Diseases Centre, National Institute of Public Health, Ljubljana, Slovenia; 2 Department of Infectious Diseases and Febrile Conditions, General Hospital Celje, Celje, Slovenia; 3 Department for Infectious Diseases and Febrile Conditions, University Medical Centre Maribor, Maribor, Slovenia; 4 Department for Infectious Diseases and Febrile Conditions, University Medical Centre Ljubljana, Ljubljana, Slovenia; 5 The members of the EPISARI Network are listed under Investigators

**Keywords:** severe acute respiratory infection, COVID-19, surveillance, Slovenia

## Abstract

We monitored trends of severe COVID-19 morbidity in Slovenia during weeks 13 to 37 2021. National weekly rates of severe acute respiratory infections (SARI) cases testing positive for SARS-CoV-2 at admission in all hospitals varied between 0.2 and 16.3 cases per 100,000 population. Of those without previous COVID-19 diagnosis, SARI COVID-19 admission rates ranged between 0.3 and 17.5 per 100,000 unvaccinated, and 0.0 and 7.3 per 100,000 fully vaccinated individuals. National SARI COVID-19 surveillance is essential in informing COVID-19 response.

The Slovenian National Institute of Public Health (NIJZ), in collaboration with all 29 Slovenian hospitals, developed surveillance of severe acute respiratory infections (SARI), known as EPISARI [1], within the comprehensive surveillance of coronavirus disease (COVID-19). By April 2021, the national EPISARI Network was established, with EPISARI contact points from all Slovenian hospitals and the EPISARI team at the NIJZ. The main objective was to monitor the national weekly numbers of SARI cases testing positive for severe acute respiratory syndrome coronavirus 2 (SARS-CoV-2) at admission to hospitals and intensive care units (ICU). In addition, we monitored the weekly numbers of (i) all SARI cases, (ii) SARI cases tested for SARS-CoV-2 infection, (iii) in-hospital COVID-19 deaths of SARI COVID-19 cases, (iv) patients with COVID-19 as a secondary diagnosis at admission, COVID-19 cases diagnosed during hospitalisation, either (v) community-acquired or (vi) healthcare-associated, and among individuals without previous COVID-19 diagnosis, (vii) SARI COVID-19 cases among individuals who were fully vaccinated or (viii) unvaccinated against COVID-19. We present selected results for the period from week 13 to week 37 2021 to demonstrate the potential of EPISARI to guide an informed timely response to COVID-19.

## Case definitions and other definitions 

A SARI case was defined as any case of acute respiratory infection of such severity to result in hospital admission. SARI COVID-19 case was defined as a SARI case with a positive SARS-CoV-2 reverse transcription PCR (RT-PCR) or antigen test result at admission [2]. COVID-19 as a secondary diagnosis at admission was defined as a patient with a positive SARS-CoV-2 RT-PCR or antigen test result at admission and without symptoms or signs of SARI. Of note, the discrimination between community-acquired and healthcare-associated COVID-19 cases diagnosed during hospitalisation was at the discretion of the EPISARI contact points at the hospitals, who were made aware of respective European Centre for Disease Prevention and Control (ECDC) surveillance definitions for COVID-19 [3].

Those with a previous COVID-19 diagnosis were defined as individuals with a record of a positive SARS-CoV-2 RT-PCR in the national COVID-19 dataset more than 3 weeks before the week under observation. Fully vaccinated individuals were defined as individuals who had received two doses of Comirnaty (BNT162b2 mRNA, BioNTech-Pfizer, Mainz, Germany/New York, United States (US)) or Spikevax (mRNA-1273, Moderna, Cambridge, US) or Vaxzervia (ChAdOx1 nCoV-19, Oxford-AstraZeneca, Cambridge, United Kingdom) or one dose of Janssen vaccine (Ad26.COV2-S, Janssen-Cilag International NV, Beerse, Belgium) at least 14 days before the week under observation. Unvaccinated individuals were defined as individuals who had not received any dose of a vaccine against COVID-19.

## Data collection 

In accordance with the national law on additional interventions for diminishing COVID-19 impact [1] and with the EPISARI objectives, all hospital EPISARI contact points reported weekly EPISARI data to NIJZ. A week was defined as Monday at 00:00 to Sunday at 24:00. While the data collection procedures were at the discretion of each hospital, completeness of reporting was 100%. Unique identifiers with information on age and sex were reported for each COVID-19 case, according to the law on healthcare datasets [4]. 

We estimated select EPISARI indicators by using data extracted from the Slovenian Central Population Registry (CPR) on 1 January 2021 as denominators. The unique identifiers were used to ascertain the vaccination status of SARI COVID-19 cases from the national electronic registry of vaccinated individuals and adverse events following vaccinations (eRCO), as well as to ascertain the previous diagnosis of COVID-19 from the national COVID-19 dataset [5]. 

## Calculating rates of SARI COVID-19 cases among fully vaccinated or unvaccinated individuals

Among individuals without previous COVID-19 diagnosis, we estimated the weekly rates of SARI COVID-19 cases per 100,000 fully vaccinated or unvaccinated individuals. Weekly numbers of fully vaccinated individuals were obtained from eRCO, while weekly numbers of unvaccinated individuals were estimated by subtracting the number of individuals who had received at least one dose of any vaccine against COVID-19 from the respective number of individuals in the CPR on 1 January 2021. To obtain the denominator for fully vaccinated individuals without previous COVID-19 diagnosis, the weekly numbers of fully vaccinated individuals with previous COVID-19 diagnosis were subtracted from weekly numbers of fully vaccinated individuals. To obtain the denominator for unvaccinated individuals without previous COVID-19 diagnosis, the weekly numbers of unvaccinated individuals with previous COVID-19 diagnosis were subtracted from weekly numbers of unvaccinated individuals.

## EPISARI weekly trends

**Figure 1:**
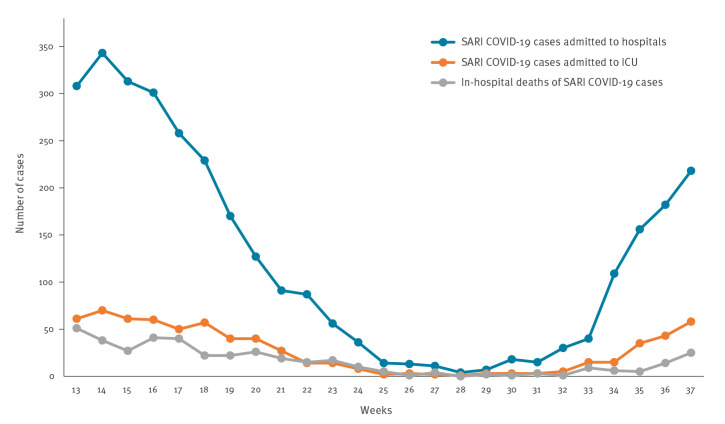
SARI COVID-19 cases^a^ admitted to hospitals and intensive care units and in-hospital deaths of SARI COVID-19 cases, Slovenia, weeks 13 to 37 2021

COVID-19: coronavirus disease; ICU: intensive care units; SARI: Severe acute respiratory infections; SARS-CoV-2: severe acute respiratory syndrome coronavirus 2.

^a^ SARI COVID 19 cases were defined as all SARI cases testing positive for SARS-CoV-2 at admission to hospitals and ICU.

During weeks 13 to 37 2021, the national weekly SARI rates varied between 7.3 and 24.1 per 100,000 population. The proportion of SARI cases tested for SARS-CoV-2 infection ranged from 91% to 100%. We identified trends in weekly numbers of SARI COVID-19 cases admitted to hospitals and ICU as well as in-hospital deaths of SARI COVID-19 cases (Figure 1). Corresponding national weekly rates for SARI COVID-19 cases ranged between 0.2 and 16.3 per 100,000 population. Weekly rates for SARI COVID-19 cases admitted to ICU ranged between 0.0 and 3.3 per 100,000 population. Weekly in-hospital death rates of SARI COVID-19 cases ranged between 0.0 and 2.4 per 100,000 population.

Data source: EPISARI surveillance of severe acute respiratory infections within comprehensive COVID-19 surveillance.

**Figure 2:**
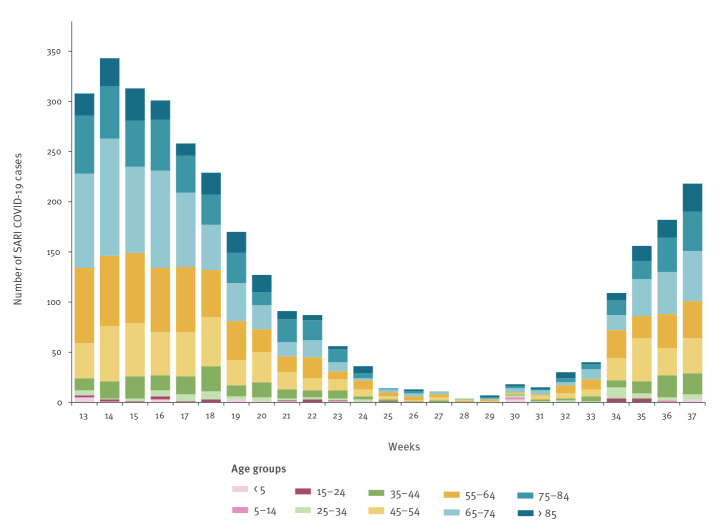
SARI COVID-19 cases^a^ by age group, Slovenia, weeks 13 to 37 2021

COVID-19: coronavirus disease; SARI: Severe acute respiratory infections; SARS-CoV-2: severe acute respiratory syndrome coronavirus 2.

^a^ SARI COVID 19 cases were defined as all SARI cases testing positive for SARS-CoV-2 at admission to hospitals.

Trends in age distribution in weekly numbers of SARI COVID-19 cases were examined according to age (Figure 2).

Data source: EPISARI surveillance of severe acute respiratory infections within comprehensive COVID-19 surveillance.

**Figure 3:**
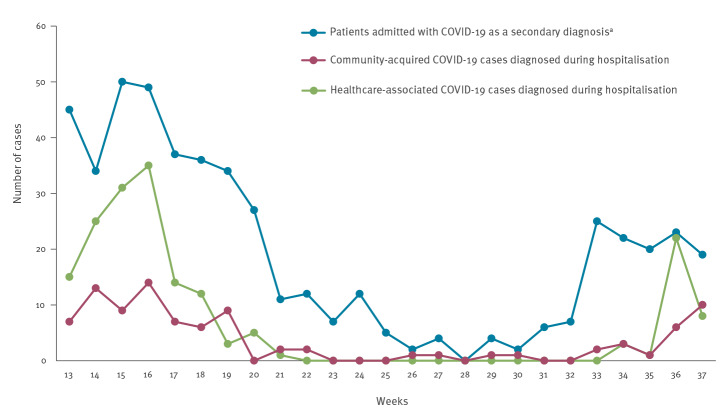
Patients admitted with COVID-19 as a secondary diagnosis^a^, community-acquired COVID-19 cases diagnosed during hospitalisation, and healthcare-associated COVID-19 cases diagnosed during hospitalisation, Slovenia, weeks 13 to 37 2021

COVID-19: coronavirus disease; SARI: Severe acute respiratory infections.

^a^ Patients admitted with COVID-19 as a secondary diagnosis were not diagnosed with SARI upon admission.

We examined trends in weekly numbers of admissions with COVID-19 as a secondary diagnosis at hospital admission as well as community-acquired and healthcare-associated COVID-19 cases diagnosed during hospitalisation (Figure 3). Weekly rates for admissions with COVID-19 as a secondary diagnosis ranged between 0.0 and 2.4 per 100,000 population. Weekly rates for community-acquired COVID-19 cases diagnosed during hospitalisation ranged between 0.0 and 0.7 per 100,000 population. Weekly rates for healthcare-associated COVID-19 cases diagnosed during hospitalisation ranged between 0.0 and 1.7 per 100,000 population.

Data source: EPISARI surveillance of severe acute respiratory infections within comprehensive COVID-19 surveillance.

**Figure 4:**
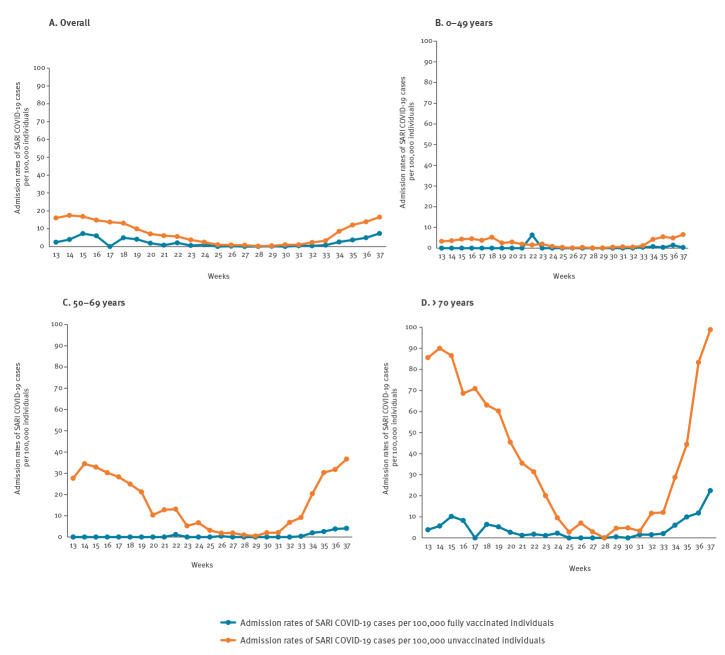
Admission rates of SARI COVID-19 cases^a^ among unvaccinated individuals without previous COVID-19 diagnosis and among individuals fully vaccinated against COVID-19 without previous COVID-19 diagnosis, (A) overall and (B),(C),(D) according to age groups, Slovenia, weeks 13 to 37 2021

COVID-19: coronavirus disease; SARI: Severe acute respiratory infections; SARS-CoV-2: severe acute respiratory syndrome coronavirus 2.

^a^ SARI COVID-19 cases were defined as all SARI cases testing positive for SARS-CoV-2 at admission to hospitals.

For individuals without previous COVID-19 diagnosis, we determined trends in the weekly rates of SARI COVID-19 cases among unvaccinated and fully vaccinated individuals, both overall and for age groups under 50 years, 50–69 years and 70 years and above (Figure 4). Overall national weekly rates for SARI COVID-19 cases among those unvaccinated ranged between 0.3 and 17.5 per 100,000 unvaccinated individuals. Respective rates among those fully vaccinated ranged between 0.0 and 7.3 per 100,000 fully vaccinated individuals. Weekly rates among unvaccinated and fully vaccinated individuals differed between the age groups examined. Of those 70 years and older, weekly rates for SARI COVID-19 cases ranged between 0.0 and 98.9 per 100,000 unvaccinated individuals and between 0.0 and 22.5 per 100,000 fully vaccinated individuals.

Fully vaccinated individuals were defined as individuals who had received two doses of Comirnaty (BNT162b2 mRNA, BioNTech-Pfizer, Mainz, Germany/New York, US) or Spikevax (mRNA-1273, Moderna, Cambridge, US) or Vaxzervia (ChAdOx1 nCoV-19, Oxford-AstraZeneca, Cambridge, United Kingdom) or one dose of Janssen vaccine (Ad26.COV2-S, Janssen-Cilag International NV, Beerse, Belgium) at least 14 days before the week under observation. Unvaccinated individuals were defined as individuals who had not received any dose of a vaccine against COVID-19.

Data source: EPISARI surveillance of severe acute respiratory infections within comprehensive COVID-19 surveillance.

## Ethical statement

Ethical approval was not necessary because EPISARI is a mandatory national surveillance system according to the law [1].

## Discussion

The magnitude of severe COVID-19 morbidity continues to be a key factor for timely and informed public health response. SARI surveillance was recommended as one of the approaches to enhance comprehensive COVID-19 surveillance by ECDC and the World Health Organization [6,7]. Currently, ECDC collects and publishes data on weekly rates of new COVID-19 hospital admissions as well as admissions to ICU or high-dependency units [8,9]. Monitoring national trends with weekly numbers or rates of SARI COVID-19 cases is essential in the context of evolving epidemic waves and changing vaccination coverages. Indeed, the rapid alterations in circulating SARS-CoV-2 variant predominance and emergence of new SARS-CoV-2 variants of concern may threaten vaccine-induced immunity against severe COVID-19 [8,10,11].

Taking into account differences in severe disease risk and vaccination coverage between different age groups of the population, as well as the fact that a substantial proportion of the population has already had COVID-19, EPISARI data will inform us about vaccine effectiveness against severe COVID-19 and waning immunity [12,13]. It should be noted that we have not yet conducted proper vaccine effectiveness analyses against severe COVID-19, but EPISARI data will make such analyses possible. Thus, our findings from EPISARI surveillance will inform our decisions on booster doses, and whether they might be indicated for everyone or only population groups of certain ages [14].

Surveillance with EPISARI has some limitations, including a variation in case ascertainment for SARI and COVID-19 between hospitals, as the data collection process is at the discretion of the individual hospital. In addition, we acknowledge possible validity issues of the data submitted to the NIJZ. Some SARI cases, as well as some COVID-19 cases, may be misclassified or under-reported. For the interpretation of data about SARI COVID-19 cases in individuals who are fully vaccinated against COVID-19, an important current limitation is lack of available information about possible immunocompromised status. Also, since not all individuals who have previously contracted COVID-19 were confirmed by a positive test, their infection would not have been registered in the national COVID-19 dataset. Therefore, we may have underestimated rates of SARI COVID-19 cases among unvaccinated and fully vaccinated individuals without a previous COVID-19 diagnosis.

## Conclusions

EPISARI is a work in progress. We are currently considering the possibility of linking the EPISARI data with whole genome sequencing data as well as investigating breakthrough SARI COVID-19 cases to elucidate possible links with risk factors for severe COVID-19. The system should also allow us to monitor the occurrence of individuals presenting as repeated SARI COVID-19 cases. In addition, we could explore the possibility to add testing of SARI cases upon admission to hospitals for other respiratory pathogens, e. g. influenza. EPISARI data are essential and robust enough to substantially contribute to an informed and timely public health response to the COVID-19 pandemic in our country.
